# Health markers, depressive symptoms, and community deprivation in a type 2 diabetes multidisciplinary care clinic for youth

**DOI:** 10.1186/s40842-024-00180-x

**Published:** 2024-07-17

**Authors:** Carolina M. Bejarano, Sanita Ley, Nisha Krishnan, Sarah Orkin, Nancy A. Crimmins, Lisa Schaaf, Amy S. Shah

**Affiliations:** 1 Division of Behavioral Medicine & Clinical Psychology, Cincinnati Children’s Hospital Medical Center, Cincinnati, OH USA; 2 Department of Pediatrics, University of Cincinnati College of Medicine, Cincinnati, OH USA; 3 Division of Endocrinology, Cincinnati Children’s Hospital Medical Center, Cincinnati, OH USA; 4 Division of Gastroenterology, Hepatology and Nutrition, Cincinnati Children’s Hospital Medical Center, Cincinnati, OH USA

**Keywords:** Type 2 diabetes, Youth, Community deprivation, Multidisciplinary care, Depressive symptoms

## Abstract

**Introduction:**

Type 2 diabetes disproportionately affects non-Hispanic/Latino Black and Hispanic/Latino youth. The purpose of this study was to examine whether differences in metabolic risk factors and depressive symptoms exist by race/ethnicity and socioeconomic deprivation and whether these impact clinic attendance and health markers over 1 year in a multidisciplinary type 2 diabetes clinic for youth.

**Methods:**

This study was a retrospective chart review of 54 youth with type 2 diabetes who had both an initial and follow-up visit. Demographic information, metabolic health markers [body mass index (BMI), hemoglobin A1C, liver enzymes, lipid panel, and urine microalbumin], depressive symptoms, and clinic attendance data were obtained from the medical record. Patient address was geocoded to the census tract level to calculate community socioeconomic deprivation.

**Results:**

Liver enzymes (ALT and AST) were significantly higher in patients identifying as Hispanic/Latino (ALT M = 97.0 ± 40.6, AST M = 53.6 ± 21.4) and lowest in patients identifying as non-Hispanic/Latino Black (ALT M = 23.1 ± 11.3, *F =* 10.6 *p < .*001; AST M = 23.1 ± 11.4, *F =* 8.1; *p < .*001) at initial visit. From initial visit to follow-up, there were significant improvements in ALT (*F* = 13.43, *p* < .001), AST (*F* = 6.58, *p* < .05), and BMIz (*F =* 18.39, *p <* .001). Patients identifying as Black or Hispanic showed an increase in depressive symptoms over time, while patients identifying as non-Hispanic White showed a decrease (*F =* 11.08; *p* < .05). Unexpectedly, patients living in areas with higher socioeconomic deprivation showed a decrease in hemoglobin A1C over time, while patients living in lower socioeconomic deprivation showed an increase (*F =* 5.15, *p <* .05).

**Conclusions:**

Differences exist in metabolic health parameters by race/ethnicity and by socioeconomic deprivation. Multidisciplinary care for youth with type 2 diabetes needs to consider and work to address the systems of inequity experienced by patients that drive disparities in health outcomes.

## Introduction

The prevalence of type 2 diabetes (T2D) and its associated comorbidities in youth continues to rise. Moreover, T2D disproportionately affects non-Hispanic/Latino Black and Hispanic/Latino youth as compared to non-Hispanic/Latino White youth [1, 2]. Previous work has found poorer hemoglobin A1c trajectories amongst non-Hispanic/Latino Black and Hispanic/Latino youth with T2D as compared to non-Hispanic/Latino White youth [3, 4]. These differences may reflect health disparities (i.e., differences between groups) driven by systems of inequity (e.g., barriers in access to care related to socioecological factors) that increase biologic risk (e.g., greater insulin secretion for the degree of insulin resistance; [5]) that disproportionately affect non-Hispanic Black and Hispanic youth. For example, structural racism contributes to unfair systems of assigning opportunity or disadvantage to individuals and communities based on social interpretation of appearance [6]. The impact of systemic inequities and social determinants of health (SDoH; i.e., the conditions in which people are born, grow, live, work, and age) are understudied in pediatric youth with T2D as compared to adult populations [7]. Social determinants of health including housing insecurity, income inequality, and poor quality neighborhood environment have been linked to increased depressive symptoms in adolescents [8]. In turn, depressive symptoms are related to increased risk for onset of T2D across the lifespan, and particularly in adolescence and young adulthood [9, 10]. Multidisciplinary care clinics are recognized as one way to help address health disparities as providers can assist with medical and psychosocial health care needs of youth and provide culturally competent care and support around diabetes self-management [11, 12]. However, as a result of social inequities, pediatric patients with T2D may visit their multidisciplinary care clinic with less frequency than recommended by their health care providers, resulting in poorer diabetes-related outcomes [13].

One way of examining social constructs which may impact health disparities is via area-level metrics such as the Community Deprivation Index (CDI). The CDI utilizes variables from US Census data related to poverty, income, education, public assistance, housing, and insurance status to generate a standardized score which in turn reflects neighborhood-level socioeconomic deprivation [14]. The use of neighborhood-level metrics such as the CDI may provide insight to the unique challenges faced by a diverse patient population and has potential to inform enhanced clinical interventions to best serve youth in a multidisciplinary pediatric T2D clinic.

The first aim of this study was to examine health disparities in metabolic risk factors and depressive symptoms by race/ethnicity (Aim 1a) and socioeconomic deprivation (as measured by the CDI; Aim 1b) in a multidisciplinary T2D clinic for youth. The second aim of this study was to determine whether disparities by race/ethnicity and socioeconomic deprivation impact metabolic health markers and depressive symptoms at one year of follow up (Aim 2a), as well as comparing the number of completed follow up clinic visits (i.e., one follow up visit vs. 2–3 follow up visits; Aim 2b). The following hypotheses were stated: (1) Racial/ethnic and socioeconomic disparities will emerge explaining the metabolic health markers cross-sectionally and at one year follow up and be reflected in the number of follow up clinic visits. (2) Higher socioeconomic deprivation as measured by the CDI will be associated with higher: hemoglobin A1C, BMI, liver enzymes, lipid panel components, urine microalbumin, and depressive symptoms and be reflected in clinic attendance.

## Methods

### Participants

This study was a retrospective chart review of youth with T2D, approved by the institutional review board at Cincinnati Children’s Hospital Medical Center (CCHMC). Patients with T2D were diagnosed using the American Diabetes Association (ADA) criteria [4] and had undetectable islet cell antibody titers. Patients included in this study were seen for an initial visit appointment between March 2018 through March 2019 and had at least one follow up visit through March 2020 to exclude the impact of the COVID-19 pandemic (i.e., last follow up visit date was March 2, 2020). Patients with other forms of diabetes (e.g., type 1 diabetes, steroid induced, or resulting from pancreatectomy) were excluded from the study. This resulted in a final sample of 54 patients. The T2D Multidisciplinary Care Clinic (MDC) is a single-location clinic that provides access to sub-specialists, referrals, and resources that best support lifestyle medication and adherence to individual T2D regiments. The clinic model has been described in more detail by Schaaf et al. [15].

### Measures

#### Patient demographics

Patient’s date of birth, age, sex, self-reported race and ethnicity, height, weight, BMI, and date of diabetes diagnosis were extracted from the electronic medical record. Type of insurance (public vs. private) was also extracted.

#### Glucose management & health markers

The following metabolic health markers were extracted for respective visits, as was available: hemoglobin A1C, aspartate aminotransferase (AST), alanine aminotransferase (ALT), gamma-glutamyl transferase (GGT), lipid profiles (total cholesterol, low density lipoprotein (LDL-C), high density lipoprotein (HDL-C), triglycerides), and urine microalbumin. Values closest to the first and one year visit were utilized (± 90 days of the clinic visit). A1C was typically assessed at the time of the visit, but assessment of other markers occurred on more varied timelines across patients.

#### Depressive symptoms

Patients self reported depressive symptoms on the Patient Health Questionnaire-9 (PHQ-9) which is a measure with strong psychometric properties validated for use in patients 12 years of age and older to assess depressive symptoms in clinical settings [16]. The measure has patients report their experience of depressive symptoms over the past two weeks on a scale ranging from 0 to 3, with higher scores indicating more depressive symptoms. The PHQ-9 is routinely completed as part of clinical care at each multidisciplinary T2D clinic, and results were extracted from the medical record. The PHQ-9 is scored with the following cut-offs for clinical use: 5 = mild, 10 = moderate, 15 = moderately severe, 20 = severe depressive symptoms.

#### Community deprivation index

The patient’s home address at the initial visit was used to determine the census tract of residence. This in turn allowed for the linkage to publicly available data from national and local databases. Six metrics from these databases are compiled to make the composite score of the CDI: (1) the fraction of households with income below poverty level; (2) the median household income in inflation-adjusted dollars; (3) the fraction of population aged 25 years and older with education of at least high school graduation or equivalency; (4) the fraction of population with no health insurance coverage; (5) the fraction of households receiving public assistance income, food stamps or supplemental nutrition assistance program; and (6) the fraction of houses that are vacant. The CDI ranges from 0 to 1 with higher values indicating greater neighborhood and sociodemographic deprivation. A median split of the range of CDI scores was used for the present analyses and is here forward referred to as *high vs. low socioeconomic deprivation*, aligned with previous studies examining CDI [17]. The software program is HIPAA compliant and has been described elsewhere [18].

### Procedures

Demographic, anthropometric, and lab data for all included participants were collected retrospectively from the electronic medical record from the time of initial visit and time of follow up. Labs were only extracted if obtained within 90 days of clinic visit. Each patient’s home address at the time of the initial visit was obtained from the electronic medical record. The offline, HIPAA compliant, open-source program ([14, 18] was used to calculate the Nationwide Community Deprivation Index by geocoding the address to the census tract level, which then enabled collection of area-level metrics.

### Data Analysis

Analyses were conducted using SPSS. For Aim 1, descriptive statistics, frequencies, and one-way between-groups ANOVAs were conducted to examine patient race/ethnicity with metabolic health and depressive symptoms, and independent samples t-tests were conducted to examine metabolic health and depressive symptoms across patients living in areas with high vs. low socioeconomic deprivation. For Aim 2, one-way repeated measures ANOVAs were used to examine pre-post comparisons of respective outcomes at year one follow up in the multidisciplinary T2D clinic. The respective outcomes were examined for initial visits vs. follow ups, using visit type (initial visit vs. follow up) as a within subjects factor. The outcomes were then subsequently examined incorporating race/ethnicity and CDI as between subjects factors. As part of Aim 2, descriptive statistics were then run to compare demographic information for patients who were able to attend one or more follow up visits during the study timeframe as compared to those who were only able to attend one follow up visit. Four race/ethnicity categories (non-Hispanic/Latino Black, non-Hispanic/Latino White, Hispanic/Latino White, and Multiracial) were used from patients at the initial visit. For some analyses, due to small sample size, race/ethnicity categories were collapsed into (1) non-Hispanic Black, Hispanic/Latino (White or Unspecified), and Multiracial and (2) non-Hispanic/Latino White to examine variables of interest: hemoglobin A1C, BMI, liver enzymes (ALT, AST, GGT), lipids (triglycerides, HDL, LDL, total cholesterol), urine microalbumin, and depressive symptoms.

## Results

The study sample included 54 participants who completed one initial visit and at least one follow-up visit within the defined study timeframe (March 2018-March 2020). Of the 130 patients seen in the multidisciplinary clinic during this timeframe, patients were excluded if they only attended an initial visit (*n* = 47) or if the time between their initial visit and follow-up visit was greater than one year and 3 months (*n* = 29). Mean age at initial visit was 15.8 ± 2.7 years (range 10–22 years); 61.1% were female and 38.9% were male; 50% self-identified as non-Hispanic/Latino Black, 31.5% as non-Hispanic Latino White, 5.5% as Multiracial, 9.3% as Hispanic/Latino White, and 3.7% as Hispanic/Latino (unspecified); 67.9% had public insurance and 32.1% had public insurance (see Table 1). Scores on the Community Deprivation Index in this sample ranged from 0.19 to 0.69, with a mean of 0.40 and median of 0.35. Preliminary analyses indicated that participants excluded from the study due to lack of a follow-up visit had CDI levels that did not significantly differ from the participant sample (range: 0.17-0.64; mean = 0.39, median = 0.36). Lastly, 81.5% of participants attended 2 or more follow up clinic visits and 18.5% attended just one follow up visit.

**Table 1:** Participant demographics

	Initial Visit (Baseline)
	*N* = 54
Demographics	*M (± SD)* or n (%)
Age	15.8 (± 2.7)
Sex	
Male	21 (± 38.9%)
Female	33 (± 61.1%)
Race/Ethnicity	
Black, non-Hispanic	27 (± 50%)
White, non-Hispanic	17 (± 31.5%)
Multiracial (Black/White), non-Hispanic	3 (± 5.5%)
White, Hispanic	5 (± 9.3%)
Unspecified, Hispanic	2 (± 3.7%)
Insurance	
Public	36 (± 67.9%)
Private	17 (± 32.1%)
Number of Follow Up Visits	
1 follow up visit	10 (± 18.5%)
2 or more follow up visits	44 (± 81.5%)
Community Deprivation Index	.4 (± .1)

Note. Descriptives obtained from baseline clinic data. *M =* Mean; *SD =* Standard Deviation. One patient did not report insurance status. CDI = Community Deprivation Index, ranging from 0–1 with higher scores indicating higher community deprivation; calculated from address at initial visit

### Differences in health markers, depressive symptoms, and community deprivation by race/ethnicity (aim 1a)

Results of Aim 1a are presented in Table 2. One-way between groups ANOVAs indicated significant differences in ALT (*F =* 10.6 *p < .*001) and AST (*F =* 8.1; *p < .*001) across racial/ethnic groups. The liver enzymes, ALT and AST, markers of metabolic dysfunction-associated fatty liver disease (MASLD/MAFLD, i.e., previously non-alcoholic fatty liver disease, NAFLD; [19]) were highest in patients identifying as Hispanic/Latino (ALT *M* = 97.0 ± 40.6, AST *M* = 53.6 ± 21.4) and lowest in patients identifying as non-Hispanic/Latino Black (ALT *M* = 23.1 ± 11.3, AST *M* = 23.05 ± 11.4). The remaining health markers and depressive symptoms did not differ significantly across race/ethnicity, but descriptives are included here and full results can be seen in Table 2. Mean hemoglobin A1C tended to be highest in patients identifying as Multiracial (*M* = 9.4 ± 4.0) and lowest in patients identifying as non-Hispanic white (*M* = 7.8 ± 1.4). PHQ-9 scores were highest (i.e., more depressive symptoms) for patients identifying as non-Hispanic/Latino Black (M = 6.5 ± 4.9) and lowest for patients identifying as Hispanic/Latino (*M* = 5.5 ± 2.1). Socioeconomic deprivation as measured by the CDI was highest in patients identifying as non-Hispanic/Latino Black (*M = .*44 ± 0.15) and lowest in patients identifying as non-Hispanic/Latino White (*M =* 0.35 ± 0.12). Considering the limitation of small group comparisons, we also examined differences across dichotomous groups (1. Black, Hispanic/Latino, and Multiracial youth and 2. Non-Hispanic White youth). In these analyses, ALT and AST were significantly higher in those identifying as non-Hispanic/Latino White (ALT: *p* < .021; AST: *p* < .041), highlighting the importance of having examined the differences across the four groups for Aim 1.

**Table 2:** Health Markers, Depressive Symptoms, and Community Deprivation in Type 2 Diabetes Clinic by Race/Ethnicity

	non-Hispanic/Latino Black (*n* = 27)	non-Hispanic/Latino White (*n* = 17)	Hispanic/Latino (*n* = 7)	Multiracial (Black/White, *n* = 3)		
Health Markers^a^	*M (SD)*	*M (SD)*	*M (SD)*	*M (SD)*	*F(*df)	*p*
Hemoglobin A1C (%)	8.8 (2.7)	7.8 (1.4)	8.3 (2.0)	9.4 (3.9)	0.72 (3)	.54
BMIz	2.4 (0.4)	2.6 (0.4)	2.5 (0.3)	2.3 (0.3)	1.44 (3)	.24
BMI percentile (%)	98.4 (4.1)	99.3 (0.7)	99.2 (0.4)	98.7 (0.9)	0.39 (3)	.78
**AST (unit/L)**	**18.3 (7.6)**	**46.1 (29.4)**	**53.6 (21.4)**	**52.3 (38.9)**	**8.1 (3)**	**< .001**
**ALT (unit/L)**	**23.1 (11.4)**	**75.9 (53.1)**	**97.0 (40.6)**	**--**	**10.6 (3)**	**< .001**
GGT (unit/L)	44.0 (28.3)	46.5 (36.7)	73.9 (42.9)	28.0 (2.8)	1.54 (3)	.23
Cholesterol (mg/dL)	151.7 (45.9)	196.8 (110.5)	175.0 (43.6)	161.3 (2.5)	1.12 (3)	.35
LDL-C (mg/dL)	90.5 (30.9)	99.3 (31.6)	104.0 (34.5)	91.7 (3.5)	0.44 (3)	.73
HDL-C (mg/dL)	47.9 (26.7)	33.6 (7.7)	37.3 (8.3)	35.0 (2.0)	1.75 (3)	.17
Triglycerides (mg/dL)	124.8 (96.9)	494.1 (1178.7)	168.3 (53.3)	173.3 (9.1)	0.92 (3)	.44
Urine microalbumin (mg/g)	36.8 (63.0)	34.8 (52.8)	10.3 (1.6)	44.4 (58.4)	0.29 (3)	.83
Depressive Symptoms^b^						
PHQ-9 Score	6.5 (4.9)	6.5 (5.5)	5.5 (2.1)	--	.04 (2)	.24
Community Deprivation Index^c^	.44 (.15)	.35 (.12)	.37 (.16)	.39 (.14)	1.64 (3)	.19

Note. Significant differences across racial/ethnic groups are bolded. *M =* Mean; *SD =* Standard Deviation. 2 of the patients identifying as Hispanic also identified as White, and 3 did not specify race. ^a^The following were missing for health markers at the initial visit: AST, *n* = 8; ALT, *n* = 8; GGT, *n* = 21; LDL, *n* = 10; HDL, *n* = 9; Cholesterol, *n* = 9; Triglycerides, *n* = 9; Urine microalbumin, *n* = 15. ^b^Depressive symptoms reflect data available for *n* = 19 patients for the PHQ-9 at the initial visit. No patients identifying as multiracial completed the PHQ-9. ^c^CDI = Community Deprivation Index, ranging from 0–1 with higher scores indicating higher community deprivation; calculated from address at initial visit. Two decimal places were retained for presentation of the CDI to most accurately present levels descriptively. Clinical cutoffs for health markers:

BMI percentile: >85% (overweight), > 95% (obesity) elevated; Hemoglobin A1C > 6.5% elevated; <6.4 normal; AST > 25 unit/L elevated; ALT > 25 unit/L elevated; LDL-C < 130 mg/dL normal; HDL-C > 40 mg/dL normal; Cholesterol < 200 mg/dL normal; Triglycerides < 150 mg/dL normal; Urine microalbumin < 30 mg/g normal

### Differences in health markers and depressive symptoms by socioeconomic deprivation (aim 1b)

Results of Aim 1b are presented in Table 3. There were no significant differences between high and low levels of socioeconomic deprivation (based on CDI) on metabolic health markers or depressive symptoms, but average levels for the sample are described here. Mean hemoglobin A1C, GGT, HDL cholesterol, and urine microalbumin tended to be higher in patients living in areas with higher levels of socioeconomic deprivation. Depressive symptoms tended to be lower for patients living in areas with higher socioeconomic deprivation.

**Table 3:** Health Markers and Depressive Symptoms Across High and Low Community Deprivation

	Low CDI^c^ (*n =* 27)	High CDI^c^ (*n =* 27)		
Health Markers^a^	*M (SD)*	*M (SD)*	*t(*df)	*p*
Hemoglobin A1C (%)	7.9 (1.7)	8.9 (2.8)	-0.92 (52)	.15
BMIz	2.5 (0.3)	2.5 (0.4)	0.06 (51)	.53
BMI percentile (%)	99.2 (0.6)	98.5 (4.1)	0.75 (51)	.35
AST (unit/L)	39.0 (28.4)	30.8 (22.9)	8.22 (44)	.29
ALT (unit/L)	58.9 (49.1)	48.7 (42.3)	10.30 (44)	.45
GGT (unit/L)	45.5 (31.1)	55.3 (40.0)	-09.78 (31)	.44
LDL-C (mg/dL)	100.0 (26.9)	90.2 (33.6)	09.76 (42)	.29
HDL-C (mg/dL)	38.0 (11.9)	44.4 (26.2)	-06.38 (43)	.29
Cholesterol (mg/dL)	181.4 (87.3)	156.9 (49.0)	24.46 (43)	.26
Triglycerides (mg/dL)	332.9 (908.1)	154.5 (92.0)	178.48 (43)	.37
Urine microalbumin (mg/g)	18.7 (26.9)	49.9 (70.3)	-31.16 (37)	.08
Depressive Symptoms^b^				
PHQ-9 Score	6.5 (5.8)	6.7 (3.8)	.14 (17)	.95

*Note. M =* Mean; *SD =* Standard Deviation. High and Low Community Deprivation Index (CDI) scores, or socioeconomic deprivation, based on median split. Scores on the Community Deprivation Index ranged from 0.19-0.69, with a mean of 0.40 and median of 0.35. ^a^The following were missing for health markers at the initial visit: AST, *n* = 8; ALT, *n* = 8; GGT, *n* = 21; LDL, *n* = 10; HDL, *n* = 9; Cholesterol, *n* = 9; Triglycerides, *n* = 9; Urine microalbumin, *n* = 15. ^b^Depressive symptoms reflect data available for *n* = 19 patients for the PHQ-9 at the initial visit. ^c^CDI = Community Deprivation Index, ranging from 0–1 with higher scores indicating higher community deprivation; calculated from address at initial visit

### Differences in health markers and depressive symptoms from initial visit to follow up (aim 2a)

Results of Aim 2a are presented in Table 4. At the initial visit the mean hemoglobin A1C of the clinic cohort was 8.4 ± 2.4 and was 8.6 ± 2.8 at follow up. The majority of patients (> 72%) had depressive symptoms in the “mild” range at both the initial visit and follow up.

Results of repeated measures ANOVAs examining health markers at the initial visits vs. follow ups indicated there were statistically significant improvements in ALT and AST liver enzyme levels (i.e., markers of fatty liver disease) between initial visits and follow ups within the multidisciplinary T2D clinic, (ALT: *F* = 13.43, *p* < .001; AST: *F* = 6.58, *p* < .05) with ALT: *M =* 53.8, *SE =* 6.7; AST: *M =* 34.9, *SE =* 3.8 at the initial visit to ALT: *M =* 39.4, *SE =* 4.66; AST: *M =* 28.3, *SE =* 2.59 at follow-up). Additionally, BMIz decreased significantly from initial visit (*M =* 2.5, *SE =* 0.1) to follow up (*M =* 2.34, *SE =* 0.1; *F =* 18.39, *p <* .001), as did BMI. There were no statistically significant differences on the outcomes of hemoglobin A1C, depressive symptoms, or other markers between the initial visits and the follow up visit with the T2D MDC. Descriptively, A1C and depressive symptoms were at similar levels at the initial and follow up visits.

**Table 4:** Descriptive Statistics of Health Markers and Depressive Symptoms at Initial Visit and Follow Up Visit

	Initial Visit (Baseline)	Follow Up Visit	
	*N* = 54 *M (SD)*	*N* = 54 *M (SD)*	*p*
Metabolic Health Markers^a^			
Hemoglobin A1C (%)	8.4 (2.4)	8.6 (2.8)	.71
**BMI (kg/m**^**2**^)	**42.9 (9.2)**	**41.3 (8.5)**	**.00**
**BMIz**	**2.5 (0.4)**	**2.4 (0.4)**	**.00**
BMI percentile (%)	98.9 (2.9)	98.4 (4.9)	.07
**AST (unit/L)**	**34.9 (25.8)**	**28.3 (17.6)**	**.01**
**ALT (unit/L)**	**53.8 (45.6)**	**39.4 (31.6)**	**.00**
GGT (unit/L)	50.4 (35.5)	41.7 (23.0)	.09
Total cholesterol (mg/dL)	170.0 (72.3)	173.1 (46.5)	.22
LDL-C (mg/dL)	95.3 (30.3)	102.4 (30.4)	1.0
HDL-C (mg/dL)	40.9 (19.9)	37.3 (8.4)	.54
Triglycerides (mg/dL)	249.7 (665.6)	189.2 (189.1)	.23
Urine microalbumin (mg/g)	33.9 (54.3)	38.2 (82.9)	.67
Depressive Symptoms^b^			
PHQ-9 Score	6.4 (4.6)	6.5 (7.1)	.63

*Note. M =* Mean; *SD =* Standard Deviation. Significant differences from initial visit to follow up visit are bolded. Descriptives obtained from baseline clinic data. One patient did not have height data to calculate BMI. ^a^The following were missing for health markers at the initial visit: AST, *n* = 8; ALT, *n* = 8; GGT, *n* = 21; LDL, *n* = 10; HDL, *n* = 9; Cholesterol, *n* = 9; Triglycerides, *n* = 9; Urine microalbumin, *n* = 15. The following were missing for health markers at follow-up: AST, *n* = 15; ALT, *n* = 15; GGT, *n* = 19; LDL, *n* = 19; HDL, *n* = 15; Cholesterol, *n* = 16; Triglycerides, *n* = 16; Urine microalbumin, *n* = 27. ^b^Data available for PHQ-9 for *n* = 19 patients at initial visit and *n* = 36 patients at follow up

**Fig. 1:**
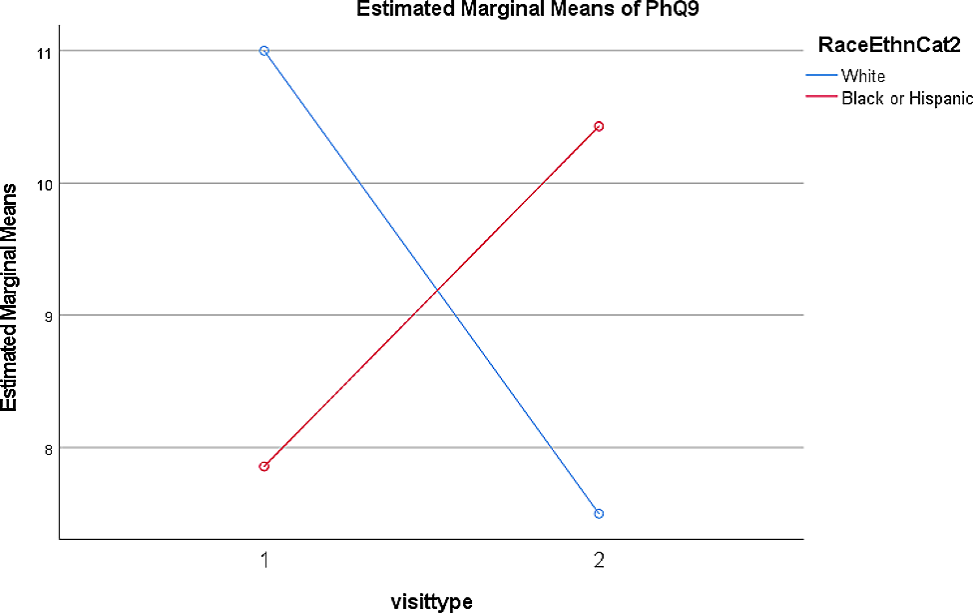
Depressive symptoms measured by the PHQ-9 at Initial Visit (1) and Follow Up (2) for groups identifying as non-Hispanic/Latino Black or Hispanic/Latino as compared to non-Hispanic/Latino White

Results of repeated measures ANOVAs examining initial visits vs. follow ups with race/ethnicity as a between subjects factor indicated there was a statistically significant difference in PHQ-9 score across race/ethnicity (*F =* 11.08; *p* < .05; see Fig. 1). In this case, patients identifying as Black or Hispanic showed an increase in depressive symptoms from the initial visit to the follow up, while patients identifying as non-Hispanic White showed a decrease in depressive symptoms from the initial visit to the follow up. Results examining initial visits vs. follow ups indicated there were no statistically significant differences on hemoglobin A1C or other metabolic health markers between the initial visits and the follow up visit with the T2D MDC, when examining or race/ethnicity as a between subjects factor.

**Fig. 2:**
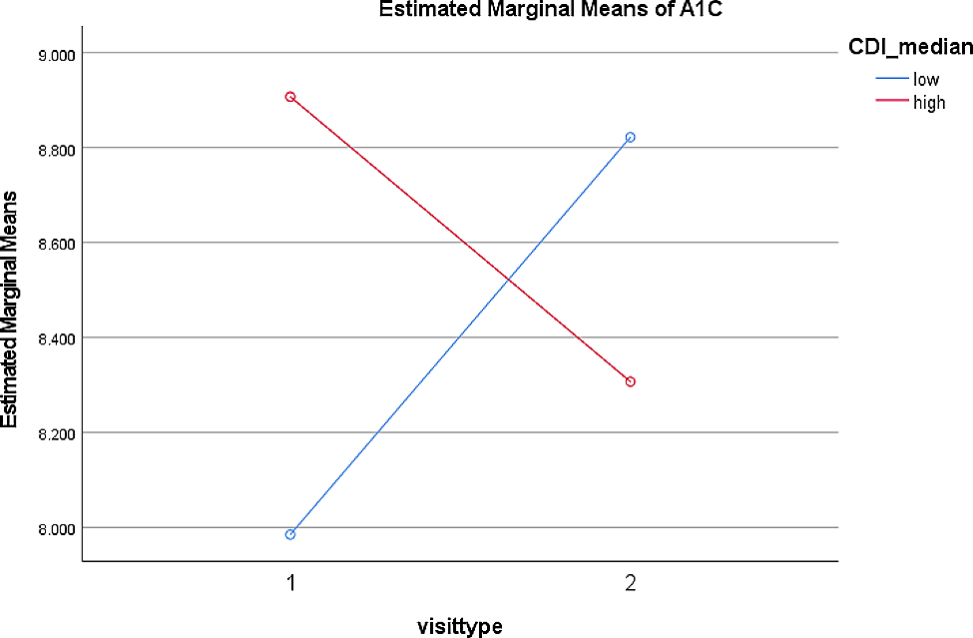
Hemoglobin A1C at Initial Visit (1) and Follow Up (2) for groups living in areas with low vs. high community deprivation scores as measured by the CDI.

Results of repeated measures ANOVAs examining initial visits vs. follow ups with socioeconomic deprivation as measured by CDI as a between subjects factor indicated there was a statistically significant difference in hemoglobin A1C across levels of socioeconomic deprivation (*F =* 5.15, *p <* .05; see Fig. 2). In this case, patients living in areas with higher socioeconomic deprivation showed a decrease in hemoglobin A1C from the initial visit to the follow up, while patients living in areas with lower socioeconomic deprivation showed an increase in hemoglobin A1C from the initial visit to the follow up. Results of repeated measures ANOVAs examining initial visits vs. follow ups indicated there were no statistically significant differences in the outcomes of A1C, depressive symptoms, or other markers between the initial visits and the follow up visit with the T2D MDC, when examining socioeconomic deprivation as a between subjects factor.

### Differences in follow up visit attendance by race/ethnicity and socioeconomic deprivation (aim 2b)

We also examined the number of follow up visits for the cohort of patients. The majority of patients (79.6%) attended 2 or more follow up visits in the study timeframe. We examined youth who attended 2 or more follow up visits by race/ethnicity and level of socioeconomic deprivation. Among those who identified as non-Hispanic/Latino Black, 81.5% had 2 or more follow up visits. Among those identifying as non-Hispanic/Latino White, 76.5% attended 2 or more follow up visits. For those identifying as Hispanic/Latino, 100% attended 2 or more follow up visits. For those identifying as Multiracial, 66.7% attended 2 or more follow up visits in the study timeframe. Additionally, patients with ≥ 2 follow up visits versus only one during the study timeframe had a significantly higher level of socioeconomic deprivation as measured by CDI score (*M =* 0.41, *SD =* 0.15) as compared to those who had only one follow up visit during the study timeframe (*M =* 0.33, *SD = .*07; *t =* 2.91, *p* < .01), which was in opposite of what was hypothesized.

## Discussion

The purpose of this study was to examine whether health disparities exist in a pediatric T2D clinic and to characterize whether metabolic and depression differences are associated with race/ethnicity or socioeconomic deprivation. Moreover, we aimed to understand the association between disparities and metabolic and depressive symptoms at one year follow up, including how often youth were able to attend clinic appointments. We found differences in metabolic health markers of ALT and AST across race/ethnicity from initial visit to follow up visit, differences in depressive symptoms across race/ethnicity and from the initial visit to follow-up, and differences in A1C across levels of socioeconomic deprivation and from initial visit to follow up visit. This suggests that that there are associations between both race/ethnicity and socioeconomic deprivation with metabolic and depressive symptoms outcomes among youth who attend our multidisciplinary T2D clinic. While we did not observe all the expected significant differences across race/ethnicity and socioeconomic deprivation as in line with previous findings [3] this may have been due to limited sample size. Descriptively in our sample, though not statistically significant, hemoglobin A1c was highest in patients identifying as Multiracial and socioeconomic deprivation was highest in patients identifying as non-Hispanic/Latino Black, while both were lowest in patients identifying as non-Hispanic Latino White.

The patient sample included here aligns with the median CDI (0.38, range: 0.12-0.85) for the county in which the multidisciplinary clinic is located (CCHMC, Hamilton County, Ohio; [18]). However, the final sample for this study was primarily non-Hispanic/Latino Black (50%) and non-Hispanic/Latino White (31.5%). While Hispanic/Latino, and Indigenous/Native American youth [1] were under-represented in this study, the sample is representative of the geographic area it serves [15] but this may have limited the potential differences in racial/ethnic groups. We found differences in liver enzymes across race/ethnicity with ALT and AST highest in patients identifying as Hispanic/Latino and lowest in patients identifying as non-Hispanic Latino Black. This finding aligns with prevalence rates of MASLD/MALFD which is partially characterized by elevated liver enzymes (ALT and AST) and affects Hispanic/Latino patients at higher rates [20] and liver enzymes tend to be lower in non-Hispanic Black youth as compared to other racial/ethnic groups [21].

We found no differences in metabolic outcomes across level of socioeconomic deprivation. These findings are fairly consistent with work examining CDI in a multidisciplinary clinic addressing metabolic health. Orkin et al. [22] found similar rates of T2D and metabolic risk factors across a range of socioeconomic deprivation, with no significant differences when examining high vs. low socioeconomic deprivation. It is possible that neighborhood-level measures obscure individual level differences and that additional metrics are needed to accurately represent socioeconomic exposures to patients that impact metabolic health and depressive symptoms. Therefore, examining CDI scores in conjunction with other measures of social determinants of health, such as food and nutrition security and access to safe spaces to be physically active [23], are an important focus of research that has been conducted in adults [24, 25] but less so in youth.

Findings indicated that there were improvements in the liver enzymes (ALT and AST) and body weight (BMIz) from the initial to the follow up appointment, but without improvements in other health markers. This may be attributable to the fact that measurable improvements in liver enzymes may more quickly be impacted by lifestyle changes, such as improvements in diet, physical activity and BMI. When examining changes from initial to follow up visit across race/ethnicity, differences in depressive symptoms emerged indicating that patients identifying as non-Hispanic/Latino Black or Hispanic/Latino experienced increases in depressive symptoms over time while non-Hispanic White patients experienced decreases in symptoms. Speculatively, racial/ethnic discrimination experienced by youth could contribute to increased depressive symptoms over time, regardless of the care and resources they may receive in their healthcare settings [8]. This is concerning, especially as depressive symptoms have shown to be a risk factor for developing T2D from adolescence to adulthood [26, 27].

When examining changes from the initial visit to follow up across levels of socioeconomic deprivation, a significant finding emerged indicating patients living in areas with higher socioeconomic deprivation experienced improved A1C (as indicated by a decreased value) from the initial visit to follow up, while patients living in areas with lower socioeconomic deprivation experienced worsening A1C (as indicated by an increased value). This is contrary to expectations that socioeconomic deprivation as a social determinant of health would contribute to inadequate glucose management and be related to higher A1C, rather than lower [23] It is possible some of the existing clinic interventions that help to address barriers to social determinants of health, such as providing transportation to clinic and facilitating access to diabetic medications influenced these results [15].

Lastly, results pertaining to the number of follow up visits were also unexpected in the sense that patients living in areas of higher socioeconomic deprivation attended more follow up visits during the study timeframe. One speculation and area to explore in future research related to this finding pertains to the higher rates of T2D in Black and Hispanic/Latino youth. Considering intergenerational risk for T2D [28], it is possible that some patients come from families with parents who also have T2D and are more familiar with the severity of the disease and the importance of implementing changes to improve their child’s outcomes. Similar to above, since the multidisciplinary clinic aids in transportation it is possible youth with higher socioeconomic deprivation are using these services to attend more visits.

### Strengths & limitations

There are limitations of this study. First, the CDI measurement was time-bound to the initial visit clinic encounter. Therefore, if youth moved home during the initial and follow visit or youth spend time in more than one household we did not examine this. Second, we lacked current medications and changes in medications over time that could explain changes or lack of changes in metabolic health outcomes. Medications were not abstracted since we could not confirm prescriptions filled or adherence to the medications listed in the medical record at the time of each respective clinic encounter. Finally, the small sample size may limit ability to detect statistically significant changes in metabolic health markers and depressive symptoms over time. Moreover, the small sample also limited the racial/ethnic grouping determinations for statistical analysis. We note that patients are typically recommended and scheduled for follow-ups with the MDC every 3–4 months, in accordance with ADA guidelines [11]. However, cancellations and missed appointments are common and contributed to the limited sample size in this study. The MDC has put several strategies in place to increase clinic attendance in ways that best meet patient and family needs [15]. Future research should continue to examine larger diverse samples that reflect prevalence rates in groups affected by T2D. The strengths of this study include the ability to examine health outcomes in terms of both race/ethnicity and social determinants of health outcomes in a population of youth with T2D which has not previously been done.

### Future direction

Multidisciplinary care for youth with T2D will continue to improve with attention to disparities experienced by patients and understanding of the systems of inequity that drive them. Continued research in this area can continue to inform standards of care [4] and impactful strategies T2D care teams can implement [15]. While this study included a robust measure of socioeconomic deprivation through the CDI, future studies may also consider other measurements that best capture systemic inequities and how they affect T2D care, metabolic health outcomes, and depressive symptoms. Recently, the MDC was in the process of implementing a social determinants of health screener to work towards standardizing the process of assessment patient needs and connecting them with relevant resources. It will be important to examine concordance of reported social determinants of health with socioeconomic deprivation as measured by the CDI in future studies. Additionally, strategies such as offering telehealth appointments and scheduling follow up visits in the clinic room at the end of the visit continue to be implemented to address challenges families may experience in accessing consistent T2D care [15].

## Conclusions

This study found differences in metabolic health markers and depressive symptoms by race/ethnicity and socioeconomic deprivation. Ongoing assessment of factors that drive health disparities in outcomes for youth with T2D can continue to inform best practice and strategies to improve access to care.

## Data Availability

The datasets generated and/or analysed during the current study are not publicly available due to institutional requirements but are available from the corresponding author on reasonable request.
